# In Vivo and In Silico Analgesic Activity of *Ficus populifolia* Extract Containing 2-O-β-D-(3′,4′,6′-Tri-acetyl)-glucopyranosyl-3-methyl Pentanoic Acid

**DOI:** 10.3390/ijms24032270

**Published:** 2023-01-23

**Authors:** Hamdoon A. Mohammed, Amr S. Abouzied, Salman A. A. Mohammed, Riaz A. Khan

**Affiliations:** 1 Department of Medicinal Chemistry and Pharmacognosy, College of Pharmacy, Qassim University, Buraydah 51452, Saudi Arabia; 2 Department of Pharmacognosy and Medicinal Plants, Faculty of Pharmacy, Al-Azhar University, Cairo 11371, Egypt; 3 Department of Pharmaceutical Chemistry, College of Pharmacy, University of Hail, Hail 81442, Saudi Arabia; 4 Department of Pharmaceutical Chemistry, National Organization for Drug Control and Research (NODCAR), Giza 12553, Egypt; 5 Department of Pharmacology and Toxicology, College of Pharmacy, Qassim University, Buraydah 51452, Saudi Arabia

**Keywords:** *Ficus populifolia*, Moraceae, in silico modeling, Discovery Studio^®^, COX-2 inhibitor, analgesic activity, acetic acid-induced writhing, analgesia meter

## Abstract

Natural product-based structural templates have immensely shaped small molecule drug discovery, and new biogenic natural products have randomly provided the leads and molecular targets in anti-analgesic activity spheres. Pain relief achieved through opiates and non-steroidal anti-inflammatory drugs (NSAIDs) has been under constant scrutiny owing to their tolerance, dependency, and other organs toxicities and tissue damage, including harm to the gastrointestinal tract (GIT) and renal tissues. A new, 3′,4′,6′-triacetylated-glucoside, 2-O-β-D-(3′,4′,6′-tri-acetyl)-glucopyranosyl-3-methyl pentanoic acid was obtained from *Ficus populifolia*, and characterized through a detailed NMR spectroscopic analysis, i.e., ^1^H-NMR, ^13^C-DEPT-135, and the 2D nuclear magnetic resonance (NMR) correlations. The product was in silico investigated for its analgesic prowess, COX-2 binding feasibility and scores, drug likeliness, ADMET (absorption, distribution, metabolism, excretion, and toxicity) properties, possible biosystem’s toxicity using the Discovery Studio^®^, and other molecular studies computational software programs. The glycosidic product showed strong potential as an analgesic agent. However, an in vivo evaluation, though at strong levels of pain-relieving action, was estimated on the compound’s extract owing to the quantity and yield issues of the glycosidic product. Nonetheless, the *F. populifolia* extract showed the analgesic potency in eight-week-old male mice on day seven of the administration of the extract’s dose in acetic acid-induced writhing and hot-plate methods. Acetic acid-induced abdominal writhing for all the treated groups decreased significantly (*p* < 0.0001), as compared to the control group (n = 6) by 62.9%, 67.9%, and 70.9% of a dose of 100 mg/kg (n = 6), 200 mg/kg (n = 6), and 400 mg/kg (n = 6), respectively. Similarly, using the analgesia meter, the reaction time to pain sensation increased significantly (*p* < 0.0001), as compared to the control (n = 6). The findings indicated peripheral and central-nervous-system-mediated analgesic action of the product obtained from the corresponding extract.

## 1. Introduction

Small-molecule drug discovery has given credence to the natural products reservoir of diverse structures, and random and designed synthetic templates obtained through various methods and approaches in drug discovery, including metabolomics, and at best, the SAR and QSAR methodologies [1,2]. The biogenetic evolution of higher and end-product(s) chemical classes of compounds of varied types passes through sets of structural advancements that are worked upon through several different enzyme-sets, biochemicals, and chemical transformations. The simpler chemical transformations include oxygen atom proliferation, methylation, acetylation, and, abundantly, glycosylation as the commonest forms. Considerable emphasis and interest had been devoted to the classical chemical class compounds, e.g., alkaloids, flavonoids and polyphenolics, sterols, terpenes, triterpenes, and mixed template compounds in drug discovery [3,4]. Certain examples include reserpine, podophyllotoxins, combretastatin A1, ecteinascidin 743, and paclitaxel. However, the recent emphasis has also shifted to include biogenetic precursors, metabolites, and symbiont molecules produced by the plants owing to the molecular-level interactions between the inter-dependent species.

The plants’ biogenic precursors and byproducts formed from the aberrant biogenetic routes/steps have lately aroused considerable interest in their structure and pharmacological properties, owing to the provision of structure minimizations and the expected activity elicitations of the structurally shortened molecular template from the pool of natural structures available in the plant’s sap. The smaller molecule activity predictions through in silico means, and the bioactivity validation(s) through in vitro and in vivo experimental ways, have also been among the recent approaches toward finding new bioactive chemical entities and lead molecules [5].

*Ficus populifolia*, from the family Moraceae, is a worldwide-distributed perennial plant that has been used since time immemorial. In modern times, the plant is primarily used as an ornamental species in designed landscapes, whereas wild plants have been used for a variety of medicinal purposes all over the world, particularly in temperate and tropical climatic zones [6]. *F. populifolia* is an ornamental shrub under the local names of ‘Madh’ and ‘Wadh’ [7], and is also popular in the Mediterranean region as part of the folk medicine. Arabian nomads and Bedouins use it for stomach pains, and occasionally for liver-related symptomatic disorders relief. The plant’s roots are also used for relieving throat pain and coughs [8]. In the MENA (Mideast and North Africa) regions and north-central Africa, the plant is used to ease labor pain, and is prescribed as a lactagogue by Nigerian herbalists to enhance milk production in breastfeeding mothers [9]. In the east African nations of Somalia, Eritrea, and Ethiopia, the plant is primarily used for the pain, itching, and healing of burn-wounds, while fruits and plant exudates are used as an alternative food by the Tanzanian tribes [10]. In the Indian subcontinent, the plant is considered sacred due to its medicinal values, and in the Indo-China regions, the plant is prescribed for several medicinal purposes, including inflammation, all types of pains, neck-area glandular swelling, stomatitis, ulcer, gout, and gum disorders [11]. The plant is also used in symptomatic heart-related disorders’ treatments, including heart/chest pain, asthma, and urinary tract troubles, as well as to relieve constipation. The plants have been reported to possess different bioactivities and pharmacological actions against several ailments representing several pharmacological classes that deal with the disorders, e.g., diabetes, respiratory, and central nervous system diseases, as well as a remedy for skin ailments [12,13]. In Egypt, the plant has been part of the medicine cabinet from time immemorial, and in preliminary pharmacological screenings, the plant’s extracts have shown antibacterial and anti-cancer activities [14]. The plant also possesses anti-neoplastic, anti-inflammatory, anti-convulsant, analgesic, antioxidant, acetylcholinesterase, proteolytic, and anti-amnesic properties [15]. Several secondary metabolites have been isolated from various parts of the plant and their extracted fractions. On a broader distribution scale, sterols and sterol glycosides, flavonoids, and polyphenols are among the common constituents from various *Ficus* species [16]. The presence of β-sitosterol, stigmasterol, and their glycosides have been confirmed in *F. bengalensis* [17], *F. odorata* [18], *F. benjamina* [19]*,* and *F. nota* [20]. The flavonoids, e.g., quercetin and luteolin [21], catechin, and genistein [22], were also reported from the genus.

A current literature survey of *Ficus populifolia* revealed that no chemical constituents are reported from this species. Herein, we report three known natural products, stigmasterol (1), β-sitosterol (2), β-amyrin (3), and a new compound (4) identified as a 3′,4′,6′-triacetylated glucoside derivative of 2(R)-hydroxy-3(S)-pentanoic acid, obtained for the first time from this *Ficus* species, *Ficus populifolia.* These natural products are also being reported for the first time from this Mediterranean species, *F. populifolia*. However, notwithstanding several different types of biological activities exhibited by the plant extract, the hereto-unknown compound (4) was pursued for its in silico activity predictions, together with the biological activity evaluation of the *F. populifolia* extract for its analgesic effects.

The sterols and triterpenes have been reported as potent analgesics, and extracts rich in these classes of natural products have been known for their analgesic activity [23,24,25]. Furthermore, the isolated steroids, compound 1, stigmasterol, and compound 2, β-sitosterol, as well as the triterpenoid, compound 3, β-amyrin have also been reported for their analgesic activity. For example, the stigmasterol exhibited biological properties to reduce the pain in both the early and late phases of the formalin test in a mice model [26]. Furthermore, stigmasterol and β-sitosterol have been shown to inhibit intraperitoneal acetic acid-induced chemical nociception [26,27]. Moreover, in a mouse model, β-amyrin was shown to possess peripheral analgesia and anti-inflammatory membrane stabilizing activity [28]. These compounds also showed a high binding affinity, and in silico inhibitory activity against cyclooxygenase 2 (COX-2) receptor-substrate [29,30,31]. Among the plethora of pharmacological activities, the compound (4) response, as part of the extract, to pain sensation and its relief, was taken up, as the pain relief has also been the most common biological activity, which had been exhibited by several other plant species of this genus, *Ficus* [7,8,9,11,32,33]. Pain, being an unpleasant sensation with multidimensional facets and involving multiple origins [34], has been treated with the widespread use of NSAIDs (non-steroidal, anti-inflammatory drugs), as well as opiates, which causes dependency, GI irritation, impairment of renal functions, inhibition of platelet aggregation, patient-compliance related troubles, together with drug tolerance issues. This has made it further pertinent to pursue the analgesic activity in more detail, including receptor binding studies, and studies on the drug-likeness of the structure (4).

Cyclooxygenase-2 (COX-2), for the majority of tissues, is the inducible form of the cyclooxygenases in pain, and its overexpression in dynamics and origins of pain together with the pain’s severity connection is well-known [35,36]. Toward the in silico analysis of compound (4), COX-2 bindings, ADME (absorption, distribution, metabolism, and excretion), and toxicity studies were pursued in detail. However, owing to the quantity issues, in vivo evaluations of the primal extract of the plant material, containing compound (4), were undertaken.

## 2. Results and Discussion

### 2.1. Isolation and Structure Elucidation of the Constituents

**Figure 1:**
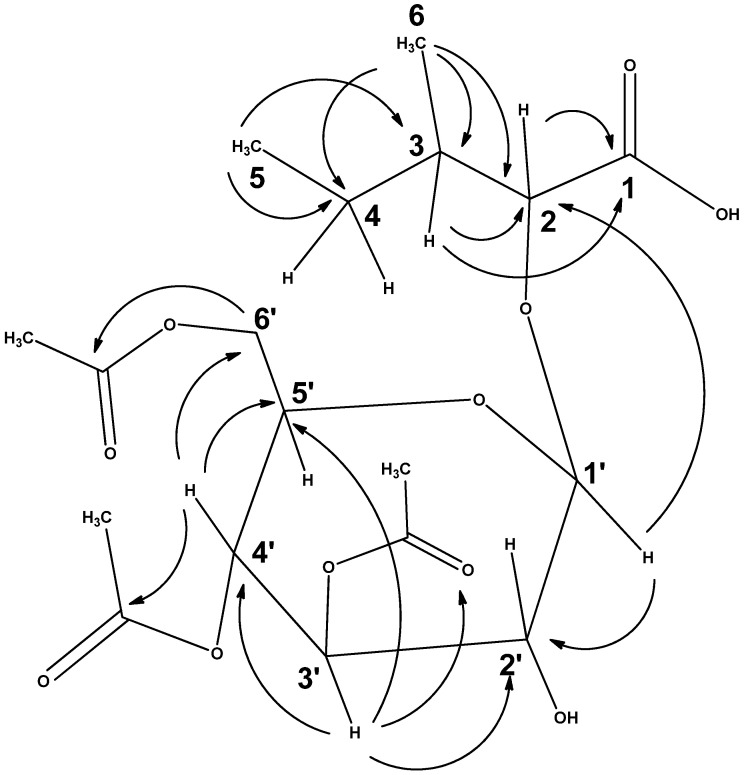
^1^H-^13^C connectivities in the structure of compound (4).

The aq.-ethanolic extract of the *F*. *populifolia* plants’ aerial parts were subjected to column chromatographic purification yielding three known and one unknown compound, stigmasterol (1), β-sitosterol (2), β-amyrin (3), and 2-O-β-D-(3′,4′,6′-tri-acetyl) glucopyranosyl-3-methyl pentanoic acid (4). All the isolated products were identified by ^1^H and ^13^C-NMR, and 2D-NMR spectral analyses. The ^1^H -NMR spectrum of compound 4 exhibited distinguishable signals for the five methyls in the compound. Two of these methyls were resonated as the most subfield signals in the H-NMR spectrum of the compound at δ_H_ 0.87 (triplet) and 0.93 (doublet), assigned to the methyls at the positions C-5 and C-6. In addition, another three downfield singlet methyls (δ_H_ 1.97, single, 2.01, s, 2.06, s) were pointing to the nature of the methyls of which one was a terminal. These methyls were assigned as part of three acetoxyl functions for which three carbonyl peaks appeared in the ^13^C-spectrum substantiated by the DEPT observations (Supplementary Materials, Figure S2). These methyls were resonated at δ_C_ 20.65, 20.55, and 20.75, and were assigned the positions 3b, 4b, and 6b, respectively, by the long-range coupling correlation (Table 1). The presence of one methylene at δ_C_ 25.55 and another methylene at δ_C_ 61.56, which were clearly observed in ^13^C and DEPT-135 NMR spectra, were assigned to the C-4 and C-6’ and indicated the aglycone and glycone natures of the methylenes, respectively. Five carbons of the methine nature at δ_C_ 68.11, 71.27, 73.20, 74.60, and δ_C_ 95.33 were assigned to the 5-methines of the sugar part of the compound, based on their chemical shift and their long coupling with their protons and the C-6’ methylene protons in the HMBC-2D NMR spectrum (Table 1). Moreover, two other methine signals appearing at δ_C_76.80 and δ_C_36.92 were found attached to protons that resonated at 4.53 (d, *J* 2.5 Hz) and 2.07 (m) in the HMQC spectrum, respectively. The positions of these two methines were assigned to be C-2 and C-3 in the aglycone part of the compound, based on their protons long coupling correlation to the carboxylic acid carbon, C-1. Furthermore, the ^13^C NMR showed four most downfield quaternary signals assigned to four carbonyl groups in the compound. Three carbonyls’ signals appearing at δ_C_ 169.67, 169.97, and δ_C_ 170.60 were attributed to the glycone, and another carbonyl appearing at δ_C_ 168.16 was elucidated to be attached to the aglycone unit. The three carbonyls for the glycone unit were shown to be acetyloxy carbonyls. The downfield shifts of methyl and methylene as substantiated by the correlation spectroscopies of compound (4) (Table 1 and Figure 1), confirmed the contention. The anomeric carbon, which was identified at δ_C_ 95.33 downfield shift, indicated the presence of a sugar moiety that was identified as the tri acetyloxy glucosyl unit while the aglycone unit was constructed to structure as 2-hydroxy-3-methyl pentanoic acid based on ^1^H and ^13^C NMR spectra, DEPT and HSQC, and HMBC correlation peaks. The ^1^H-NMR spectrum in conjunction with ^13^C-DEPT, and 2D correlations of the quaternary carbon resonances, exhibited peak at δ_C_ 168.16 ppm (COOH). Five other carbons resonating at δ_C_ 76.80 (C-OH, C-2), δ_C_ 36.92 (CH, C-3), δ_C_ 25.55 (CH_2_, C-4), δ_C_ 11.58 (CH_3_, C-5), and δ_C_ 11.96 ppm (CH_3_, C-6), along with their corresponding hydrogens chemical shift values and coupling constants for the stereo orientations of the C-2 methine (d, 2.5 Hz, showing connectivity to C-1 and C-3 carbons), and C-3 methine groups (2.07, m, showing connectivity to C-1, and C-2 carbons) were exhibited, which confirmed the 2(S)-hydroxy-3(R)-methyl pentanoic acid-derived substructure. The ^1^H-NMR spectrum also showed two methyl peaks at δ_H_ 0.87, t. *J* = 7.5 and δ_H_ 0.93, d, *j* = 7 for H-5 and H-6 protons, respectively. Additionally, the ^1^H-NMR spectrum showed one proton doublet at δ_H_ 4.53 (H-2), along with three other protons multiplets at δ_H_ 1.38, δ_H_ 1.47, and δ_H_ 2.07 for H-4b, H-4a, and H-3 protons, respectively (Table 1). The sugar moiety of compound (4) was assigned as 3′,4′,6′-tri-acetyl glucose by the presence of anomeric carbon at δ_C_ 95.33 (C-1’) and one methylene carbon at δ_C_ 61.56 (C-6’), with other four methines carbons resonating between δ_C_ 68.11 and δ_C_ 74.60. The presence of one proton doublet at δ_H_ 4.76 (H-1’) and six protons resonating between δ_H_ 3.80 and δ_H_ 5.28 also led to elucidating the sugar moiety as acetylated glucose. Additionally, three acetylated methyl singlets resonating at δ_H_ 1.97, δ_H_ 2.01, and δ_H_ 2.06 appeared in correlation with the three carbonyls at δ_C_ 169.97, δ_C_ 169.67, and δ_C_ 170.60, as shown in the HMBC spectrum (Supplementary Materials, Figures S1–S6), were found attached to the C-3′, C-4′, and C-6′ carbons, respectively.

The attachments of the acetyl carbons to the sugar were assigned according to the HMBC long-range correlations and are depicted in Figure 1, which clearly shows a correlation between δ_H_ 5.28 (H-3’) and δ_C_ 169.97 for the C-3′ (acetoxy C=O) and δ_H_ 5.04 (H-4’) with δ_C_ 169.67 for the C-4′ (acetoxy C=O), in addition to long-range coupling between δ_H_ 4.10 and δ_H_ 4.26 of H-6’ and δ_C_ 170.60 for the C-6′ (acetoxy C=O). Although partially acetylated glucose/glycone is rarely found in nature, nonetheless there are few reports of acetylated sugars, e.g., mono-acetylated allose as part of the flavonoid-glycoside structure isolated from *Stachys anisochila* [37]. However, partially acetylated glucose in structures (Figure 1) was also confirmed by the presence of three methyl singlets appearing at δ_H_ 1.97 (3′-acetoxy methyl), δ_H_ 2.01 (4′-acetoxy methyl), and δ_H_ 2.06 (6′-acetoxy methyl), in connectivity with the ^13^C-NMR spectrum showing carbons at δ_C_ 20.65, δ_C_ 20.55, and δ_C_ 20.75. Additionally, the ^13^C-DEPT (Table 1) confirmed the presence of four quaternary carbons for the previous three acetyls along with one carboxylic acid carbon resonating at δ_C_ 168.16 for the C-1 (aglycone part) carbon, which was found coupled with the proton doublet resonating at δ_H_ 4.53 (H-2) in the HMBC spectrum. The chemical shift values of the acetylated methyls revealed that these acetyl groups must be attached to the sugar moiety of compound (4) and not to the aglycone part [38]. The distinct downfield shift of the sugar protons H-3’ and H-4’, as compared to the reported values of the fastigitin-A showed protons, peaks additionally downfield by δ +1.1, and +0.86 ppm, respectively [20]. Moreover, the long-range coupling appearing in 2D analyses confirmed that the quaternary carbon at δc 169.97, 169.67, and δ_C_ 170.60 is the nodal carbon for the three acetyl groups, which are attached to the sugar carbons at C-3’, C-4’, and C-6’, respectively. The attachment of the triacetylated glucose to the aglycone part was assigned through the long-range coupling in the HMBC spectrum between the doublet proton resonates at δ_H_ 4.76 of the anomeric proton (H-1’) with the δ_C_ 76.80 for the C-2. In addition, the glycone was assigned as β-D-triacetylated glucose based on the *J*-coupling of the anomeric proton, which was found as 8.5 Hz that showed the β-linkage of the glucose to the aglycone part in parallel to the fastigitin-A, which has been reported from *Rhodiola fastigiata* [39], the significant difference being the presence of tri-acetoxy functions in the glucose unit.

Therefore, the ^1^H and ^13^C-NMR correlations and HMBC led to elucidating the structure of compound (4) as a (3*S*)-2-hydroxy-3-methyl pentanoic acid derivative with a 3′,4′,6′- triacetoxy-β-D-glucosyl unit (Figure 1).

Compound (1), stigmasterol, isolated as a white amorphous powder with a melting point of 170 °C, was identified based on spectral data, which were similar to β-sitosterol, compound (2); except for the presence of two multiple olefinic protons at δ_H_ 5.03 and δ_H_ 5.20, in addition to the presence of two olefinic carbons at δ_C_ 129.25 and δ_C_ 138.34 ppm. The structure was also confirmed through 2D-NMR spectral analyses, and literature comparisons [40,41].

Compound (2) was isolated as a white amorphous powder with a melting point of 141 °C. The IR spectrum showed absorption peaks at 1050 cm^−1^ (C-O), in addition to stretching at 3428 cm^−1^ (O-H) and 1640 cm^−1^ (C=C). The ^1^H- and ^13^C-NMR spectra showed a similar pattern to the published data of β-sitosterol. The structure was confirmed through the presence of a multiplet peak at δ_H_ 3.51 ppm (H-3), along with a double doublet at δH 2.26 for the methylene protons (H-7) and with the multiple peaks at δ_H_ 5.33 ppm for the olefinic proton (H-6). Additionally, the ^13^C-NMR showed peaks at δ_C_ 140.76 (C) and 121.85 ppm (CH) for the C-5 and C-6 carbons, respectively. The carbon signal at δ_C_ 71.81 ppm for C-3 along with another 26 aliphatic carbon signals that strongly supported the β-sitosterol structure for compound (2), which was also supported by the 2D-NMR spectral data [42,43].

Compound (3) was isolated as a pale amorphous mass with a melting point of 189 °C and identified as β-amyrin based on the NMR spectral data and in comparison with the reported values. The presence of δ_H_ 3.21 (H-3) as a double doublet and the peak at δ_H_ 5.17 (H-12) as a triplet in addition to the carbon’s signals resonating at δ_C_ 79.90 (C-3) and δ_C_ 121.750 ppm (C-12) with the quaternary carbon at δ_C_ 145.30 ppm, confirmed the β-amyrin structure. The spectral data from ^1^H and ^13^C-NMR, DEPT, HMBC, and HMQC spectra conclusively identified the β-amyrin structure [44].

### 2.2. Docking Studies for Analgesic Activity

**Figure 2:**
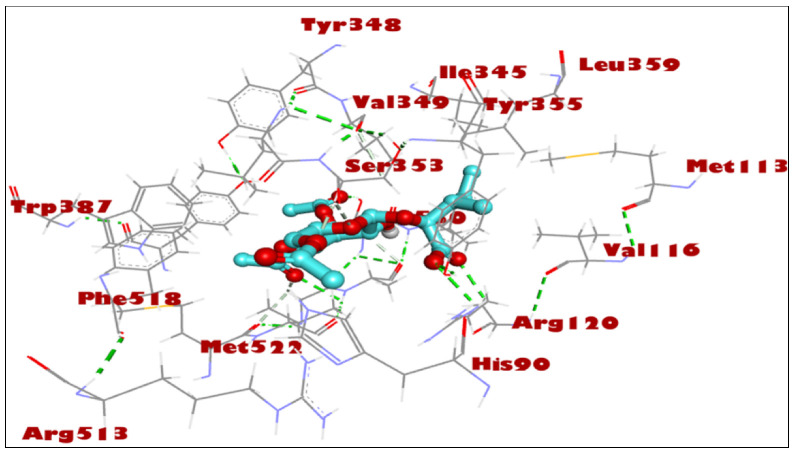
Three-dimensional molecular interactions of 3′,4′,6′-triacetylated pentanoic acid glucoside (compound 4) with human cyclooxygenase-2 (COX-2) residues. The hydrogen bonds are represented as green dotted lines.

**Figure 3:**
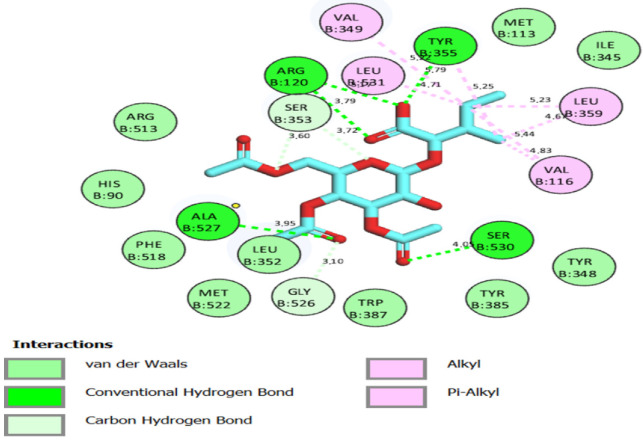
Two-dimensional molecular interactions of 3′,4′,6′-triacetylated pentanoic acid glucoside (compound 4) with human cyclooxygenase-2 (COX-2) residues.

**Figure 4:**
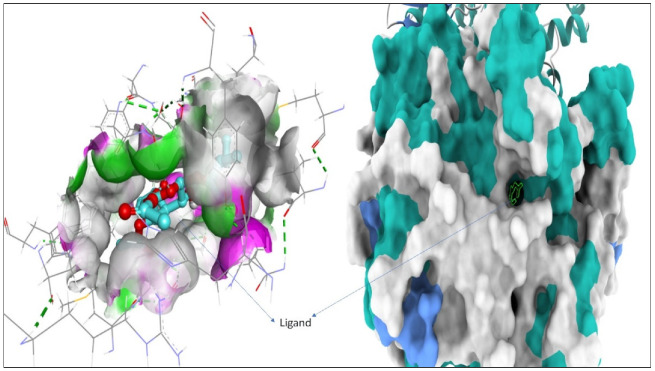
Surface mapping showing the ligand (compound 4, the 3′,4′,6′-triacetylated pentanoic acid glucoside) occupying the active pocket of the human cyclooxygenase-2 (COX-2).

For the study, the human cyclooxygenase-2 (COX-2) protein was selected. COX-2 converts arachidonic acid to prostaglandin. Prostaglandin is subsequently metabolized by the downstream tissue-specific synthases into potent signaling molecules that play fundamental roles in both the regulation of physiological homeostasis as well as in disease states such as nociceptive inflammation and cancer [45]. The compounds 1–3 are known for their potential analgesic activity [26,27,28] and have shown a high binding affinity and in silico inhibitory activity against COX-2 [29,30,31]. In this study, the binding efficiency of the 3′,4′,6′-triacetylated pentanoic acid glucoside (compound 4) was calculated. Lower binding energy, resulting from the association of the compound with the targeted protein, is an indication of a higher binding efficiency. NAG was used in this study as a COX-2 inhibitor by comparing the binding affinity of compound 4 (ΔG of −8.1) with NAG (ΔG of −7.6). It was found that compound (4) showed a very good binding affinity. These results of the in silico protein-screened metabolites interactions showed that the following amino acid in the protein target participated actively in the interactions (VAL 349, TYR 355, LEU 359, VAL 116, SER 530, ALA 527, and GLY 526) through the number of hydrogens, and followed hydrophobic interactions, Table 2, Figure 2, Figure 3 and Figure 4. The observations established the strong anti-analgesic potential of compound (4), which nonetheless, can be taken as the primary contributor of the anti-analgesic activity of the *F. populifolia* extract, though the contributions of the anti-analgesic activities of other constituents, including compounds (1), (2), and (3), may or may not be in synergistic conditions.

### 2.3. Physiochemical and ADMET Profiling

**Figure 5:**
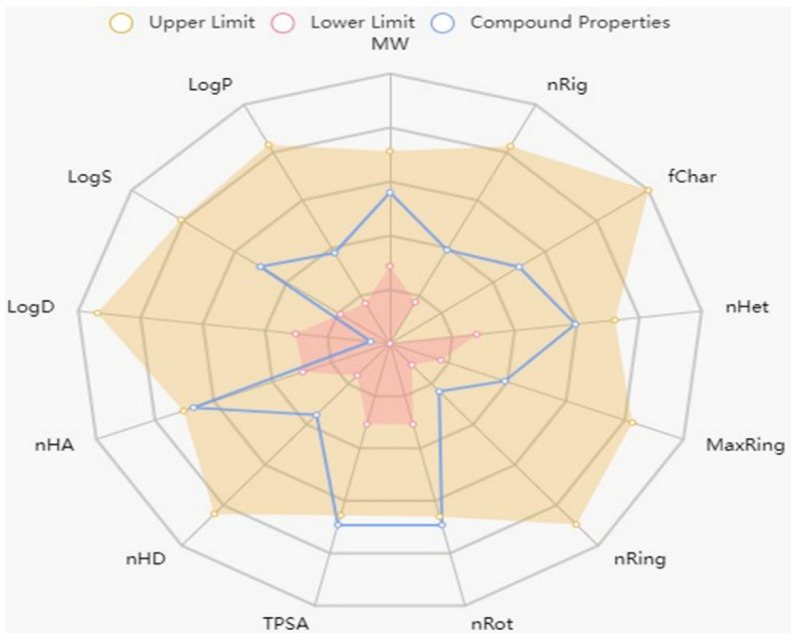
Bioavailability radar of 3′,4′,6′-triacetylated pentanoic acid glucoside (compound 4).

The drug-likeness behavior of compound (4) was estimated using the Swiss ADME program [46]. The listed data in Table 3 and Table 4 show that, compound 4, 3′,4′,6′-triacetylated pentanoic acid glucoside, has a molecular weight <500, which increased the transport and absorption and improved the transmissibility of the compound to the membranes. The topological polar surface area (TPSA) of this compound found in the correct published range is less than 140 Å^2^ (Table 3 and Table 4) [47,48]. Lipophilicity (Log P) values ranged within 1.5–4.81, which is considered compatible by Lipinski’s rule of five. The lipophilicity, which is also related to toxicity generation at a certain scale, has demonstrated that the toxicity is significantly higher for derivatives with log Po/w > 5 and TPSA < 75 Å^2^ [49]. The obtained results showed that the newly investigated compound (4) has physicochemical characteristics that are within the appropriate range, as also demonstrated by the bioavailability radar (Figure 5). Thus, the study indicated that compound (4) is non-toxic in nature and has the suitable physico-chemical and ADMET properties for being fit to be a lead compound for future development.

Table 5 depicts the toxicity data of compound (4), 3′,4′,6′-triacetylated pentanoic acid glucoside. The ADMET lab 2.0 software used the AMES toxicity test that described the potential carcinogenic effect of the compound [50] and according to the human ether-a-go-go-related gene (hERG) toxicity test, the blockage of the potassium channel of that gene leads to cardiac toxicity [51]. Furthermore, the software estimated the oral rat chronic toxicity (LOAEL), hepatotoxicity, and skin sensitization.

In addition to the toxicity model, the toxicophoric rules were also estimated for identifying the ability of compound (4) to induce cancer by mutations (genotoxic carcinogenicity rule), or by any other mechanism rather than the mutation (non-genotoxic carcinogenicity rule) [52]. These results revealed that the tested compound (4) had a non-toxicity impact in any toxic model (Table 5), and thus, proved to be of interest for further development in the drug discovery program. The predictions of the pharmacokinetics properties, with one violation involving the N or O atoms abundance which is part of the molecular framework and can be improved through use of bioisosteric group replacement in subsequent design(s), further supported the notion of the drug-likeliness of the product compound (4).

### 2.4. In Vivo Analgesic Activity Evaluations

**Figure 6:**
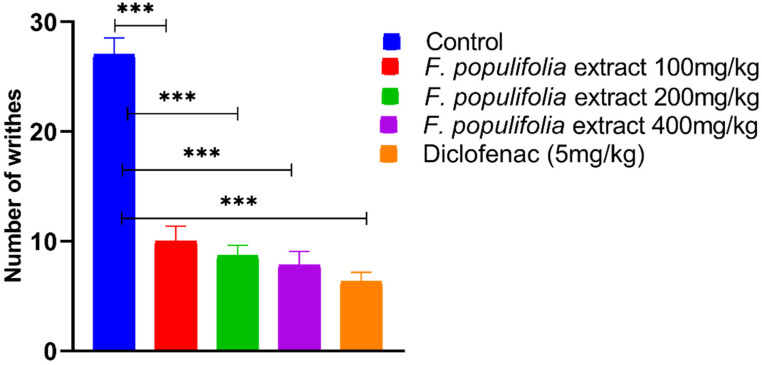
Evaluation of peripherally mediated analgesic activity using acetic acid-induced writhing model. The number of incidences of writhing for the mouse groups is expressed as mean ± standard error, n = 6 mice per group. Extract groups: total extract of *F. populifolia*. *** Data differed significantly at *p* < 0.0001 when compared with the control group in the relevant column.

**Figure 7:**
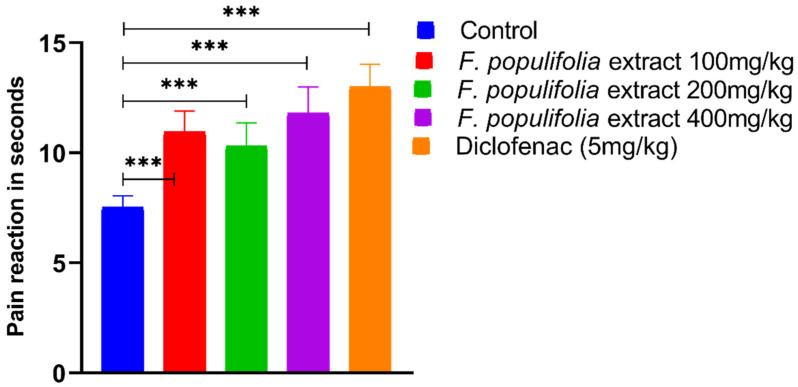
Evaluation of centrally mediated analgesic activity using the analgesia meter method. Pain reaction values expressed as mean ± standard error, n = 6 mice per group. Extract groups: total extract of *F. populifolia*. *** Data differed significantly at *p* < 0.0001 when compared with the control group in the relevant column.

Analgesic activity of the compound containing extract was undertaken since the material quantity was in adequate. Furthermore, the plant extract contains significant amounts of steroids and tetraterpenoids, which are well-known for their analgesic and anti-inflammatory properties [53,54]. As observed, the number and percentage of acetic acid induced abdominal writhing were decreased significantly, (*p* < 0.0001). It was observed for all the treated groups in comparison to the control group (27.6 ± 1.53) by 10.00 ± 1.37, 8.67 ± 0.95, 7.83 ± 1.25, 6.33 ± 0.84, and 62.9%, 67.9%, 70.9%, and 76.5% for the extract groups at 100 mg/kg, 200 mg/kg, and 400 mg/kg, and the diclofenac 5 mg/kg group, respectively (Figure 6). The reaction time to pain, measured using an analgesia meter, increased significantly (*p* < 0.0001) as compared to the control group (7.53 ± 0.95) by 10.95 ± 0.95, 10.32 ± 1.05, and 11.80 ± 1.20, and 13.00 ± 1.03 for all the extract groups 100 mg/kg, 200 mg/kg, and 400 mg/kg, and the diclofenac 5 mg/kg group, respectively (Figure 7).

The extract of the plant *F. populifolia* showed peripherally mediated analgesic activity using an acetic acid-induced writhing model. Researchers have previously described acetic acid-induced writhing [55] and the hot plate method [56] using an analgesia meter as the model for the evaluation of analgesic activity mediated at the peripheral and central levels, respectively. The peripheral level mediation is hypothesized to act through the inhibition of cyclooxygenases, especially and primarily through COX-2. Data from our current study confirmed the analgesic activity in an acetic acid-induced writhing model for the first time using the extract of the *F. populifolia* plant. The data are also in confirmation with other species of *Ficus* in their analgesic activity [57,58,59,60,61].

Pain induced by the thermal stimulus of the hot plate is specific for centrally mediated nociception [32]. The ability of the *F. populifolia* extract to increase the latency of pain reaction induced by a hot plate, described here for the first time, indicates the central analgesic potential of the extract. However, other constituents, e.g., flavonoids, steroids, and tannins are also known to have analgesic activities [59].

## 3. Materials and Methods

### 3.1. Chemistry: Plant Material, Extraction, and Isolation of Products

*Ficus populifolia* aerial parts were collected from Qassim, Saudi Arabia, in fruiting season and were authenticated by the institutional taxonomist at the College of Agriculture, Qassim University. A voucher sample number QPP-125 was kept at the College of Pharmacy, Qassim University, Saudi Arabia. The freshly collected plant material (1.1 kg) was shade-dried, grinded, and extracted over a period of 6 h cycle in a Soxhlet apparatus (BLS.2307.13) with light petroleum ether, followed by 95% aq. ethanol to yield 22 gm and 64 gm of viscous masses, respectively, after concentration on vacuum rotatory evaporator under reduced temperature and pressure. The EtOH extract was examined on the TLC (Merck, Darmstadt, Germany, catalogue number, HX99037354) and subjected to silica gel (70–230 mesh, Supelco, Tokyo, Japan, Lot MKCP3826) column chromatography (CC) (CG119720) in CHCl_3_ and CHCl_3_-MeOH (*v*/*v*) and was afforded fractions with impure products which were further purified by the Sephadex^TM^ LH-20 (GE Healthcare Bio-Sciences, Uppsala, Sweden) and silica gel column chromatography in hexane-ethyl acetate varying polarities. Of the purified products, compound (1) was identified as stigmasterol, compound (2) as β-sitosterol, and compound (3) as β-amyrin, based on their reported spectral data including 2D NMR analyses. Compound (4) was isolated as a gummy substance (CHCl_3_-MeOH, 88/12: *v*/*v*, fractions 29–33, 1 × 50 mL) and was further purified by reverse-phase silica-C_18_ (Sigma, Buchs, Switzerland, Lot BCC82847) CC using methanol–water mixture (1:0.5) as mobile phase; R_f_ 0.6, 15% MeOH-CHCl_3_, *v*/*v*; [α]_D_ (−17.2, c 1, CHCl_3_). Elemental analyses were performed on Elementar Vario EL III, Carlo Erba 1108 (Marlton, NJ, USA), Anal. C 51.54%, H 6.79%, cald for C_18_H_28_O_11_, C 51.41%, and H 6.73%; NMR (Bruker 500 MHz, TMS reference standard, CDCl_3_): Table 1.

### 3.2. Biological Activity: Animal Groups

Thirty eight-week-old male mice were obtained from the animal facility, College of Pharmacy, Qassim University. The animals were housed in polyacrylic cages and maintained at room temperature with a relative humidity of 45–65% in controlled light and dark cycles. The Research Ethics Committee, College of Pharmacy, Qassim University, Saudi Arabia, approved all the experimental procedures (21-04-06). The care of laboratory animals was carried out as per the Guide for the Care and Use of Laboratory Animals. The extract doses (100 mg/kg, 200 mg/kg, and 400 mg/kg) were selected based on the previously published article [61].

### 3.3. Evaluation of Peripherally Mediated Analgesic Activity Using Acetic Acid-Induced Writhing Model

Induction of writhing using acetic acid was performed as previously described by Koster et al. [56] method. Briefly, mice were divided into 5 groups (n = 6/group). The control group received normal saline (10 mL/kg oral route (p.o.). Groups 2–4 received 100 mg/kg, 200 mg/kg, and 400 mg/kg extract p.o. daily for one week, while the positive group received Diclofenac 5 mg/kg, i.p, on the day of the test. On the seventh day, 30 min after the last dose, 0.05% acetic acid/100 g, i.p, was injected into all the 5 groups. Five minutes after the dose, abdominal constrictions were counted for each mouse for 10 min. Data were recorded, and percentage inhibition of writhing was calculated using the formula:

$$Inhibition\;\%=\left[\frac{c-t}{c}\right]\times \;100$$

where *c* = Mean number of writhing in control group and *t* = Mean number of writhing in treated group.

### 3.4. Evaluation of Centrally Mediated Analgesic Activity Using Analgesia Meter Method

Hot plate tests were performed as described previously [25,55]. Briefly, the temperature on the analgesia meter was set to 55 ± 1 °C. The time taken by the mice for licking or jumping was recorded with 15 s as a cutoff point. All the mice were separated into five groups (n = 6/group). The control group received normal saline (10 mL/kg, p.o.), groups 2–4 received 100 mg/kg, 200 mg/kg, and 400 mg/kg extract p. o. daily for one week, while the positive group received diclofenac 5 mg/kg, i.p on the day of the test. On the seventh day, 30 min after the last administration, the mice were placed on a hot plate, and the sensitivity to pain in seconds was documented.

### 3.5. In Silico Studies: COX-2 Docking

**Figure 8:**
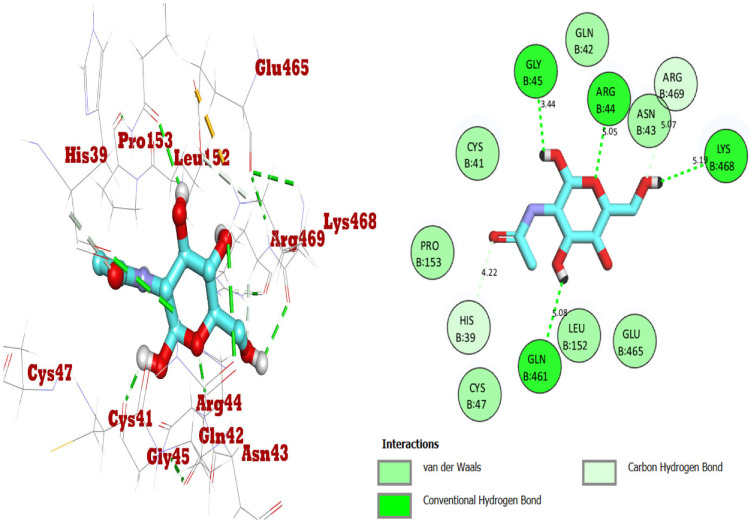
Two-dimensional and three-dimensional molecular interactions of the re-docked co-crystalized ligand (NAG) with human cyclooxygenase-2 (COX-2). The hydrogen bonds are represented as green dotted lines.

The MOE 2019.012 suite [62] was applied to carry out the docking studies for the 3′,4′,6′-triacetylated pentanoic acid glucoside to propose their mechanism of action as analgesic, through evaluating their binding scores and modes compared with NAG (2-acetamido-2-deoxy-β-D-glucopyranose) as the co-crystallized ligand. The 3′,4′,6′-triacetylated pentanoic acid glucoside was introduced into the MOE window, subjected to partial charges addition, and its energy was minimized. Then, the prepared compound was inserted into one database with NAG and saved as an MDB file to be uploaded in the ligand icon during the docking step. The X-ray crystallography of the target human cyclooxygenase-2 (COX-2) was obtained from the Protein Data Bank (https://www.rcsb.org/structure/5IKR, accessed on 25 October 2022). Moreover, it was prepared for the docking process following the previously described steps in detail. Notably, the downloaded protein was corrected for any errors, loaded with 3D hydrogens, and the was energy minimized as well. The 3′,4′,6′-triacetylated pentanoic acid glucoside was inserted into a general docking process in place of the ligand site. The docking site was chosen to be the co-crystallized ligand site and the docking process was initiated after adjusting the default program specifications described before [3]. Briefly, the dummy atoms method was used to select the docking position. Triangle matcher and London dG were selected as the placement and scoring methodologies, respectively. Both the refinement methodology and the scoring one were changed to the rigid receptor and GBVI/WSA dG, respectively, to extract the best 10 poses produced from 100 poses for the docked molecule. The best pose for the ligand with the most acceptable score, binding mode, and RMSD value was selected for further studies. It is worth clarifying that a program validation step was performed first for the applied MOE program by re-docking the co-crystallized ligand (NAG) at its binding pocket of the prepared target (Figure 8). Valid performance scores were confirmed by obtaining a low RMSD value (1.43) between the screened compound and the re-docked, co-crystallized ligand (NAG). The output from MOE software was further visualized by Discovery Studio 4.0 software.

### 3.6. Statistical Analysis

Data were reported as mean ± standard error of the mean (SE). The difference between groups was analyzed using one-way ANOVA followed by post-hoc test using Dunnett’s multi-group comparison on GraphPad Prism 8.0.2. The data were considered significant if *p* < 0.05.

## 4. Conclusions

In silico studies confirmed the analgesic potential of the isolated new compound (4), 2-O-β-D-(3′,4′,6′-tri-acetyl)-glucopyranosyl-3-methyl pentanoic acid, and the in vivo experimental data from the current study indicated the significant nociceptive potential of the *F. populifolia* extracts at all the evaluated doses. Further research is required on the presence and role confirmation of the predicted active constituent of the plant.

## Figures and Tables

**Table 1:** NMR values and correlations of compound 4.

Carbon Number	^1^H δ-Value	^13^C δ-Value	DEPT (135)	^1^H-^1^H COSY	^1^H-^13^C HMBC
1		168.16	C=O		
2	4.53, d, *J* 2.5 Hz	76.80	CH	H-3	C-1
3	2.07, m	36.92	CH	H-4a and 4b, H-2 and H-6	C-1 and C-2
4	1.47, m	25.55	CH_2_	H-3 and H-5	
	1.38, m				
5	0.87, t. *J* 7.5 Hz	11.58	CH_3_	H-4a and 4b	C-3 and C-4
6	0.93, d, *J* 7 Hz	11.96	CH_3_	H-3	C-2, C-3 and C-4
1’	4.76, d, *J* 8.5 Hz	95.33	CH	H-2’	C-2 and C-2’
2’	4.24, m (overlapped)	74.60	CH	H-1’ and H-3	
3’	5.28, t, *J* 9.5 Hz	71.27	CH	H-2’ and H-4	C-2’, 4’,5’ and C-3′ (acetoxy C=O)
4’	5.04, t, *J* 9.5 Hz	68.11	CH	H-3’ and H-5’	C-3’, 5’ 6’ and C-4′ (acetoxy C=O)
5’	3.80, m	73.20	CH	H-4’ and H-6’a and 6’b	
6’	4.26, dd, *J* 12.5, 1.5 Hz	61.56	CH_2_	H-5’ and H-6’b	C-5’ and C-6′ (acetoxy C=O)
	4.10, dd, *J* 12.5, 3.0 Hz			H-5’ and H-6’a	C-4’, 5’ and C-6′ (acetoxy C=O)
3′	(acetoxy C=O)	169.97	C=O		
4′	(acetoxy C=O)	169.67	C=O		
6′	(acetoxy C=O)	170.60	C=O		
3′	1.97, s (acetoxy methyl)	20.65	CH_3_		C-3′
4′	2.01, s (acetoxy methyl)	20.55	CH_3_		C-4′
6′	2.06, s (acetoxy methyl)	20.75	CH_3_		C-6′

**Table 2:** The binding scores and interactions of 3′,4′,6′-triacetylated pentanoic acid glucoside (compound 4) against COX-2 (PDB ID: 5IKR).

Compound	Binding Score (kcal/mol)	Hydrogen Bond Interactions	Distance (Å)	Hydrophobic Interactions	Distance (Å)
3′,4′,6′-Triacetylated pentanoic acid glucoside	−8.1	TYR 355 GLY 526 SER 530	2.34, 2.53 3.34 1.97	VAL 349 LEU 352 TYR 355 LEU 359 TRP 387 PHE 518 VAL 523 LEU 531	3.42 3.67 3.78, 3.41 3.13 3.52 3.63 3.68 3.31
(NAG)2-Acetamido-2-deoxy-β-D-glucopyranose	−7.6	ARG 44 GLY 45 GLN 461 LYS 468	2.08 2.21 2.65 2.69	CYS 41 ASN 43	3.50 3.24

**Table 3:** Physicochemical properties of compound 4.

Molecular Weight	Number of Heavy Atoms	Number of Aromatic Heavy Atoms	Number of Rotatable Bonds	Number H-Bond Acceptor (HBA)	Number H-Bond Donors (HBD)	Topological Polar Surface Area (TPSA)	Lipophilicity (Log P)	Water Solubility (Log S)
420.16	29	0.0	12	11	2	154.89	1.87	−1.741

**Table 4:** Pharmacokinetics properties of compound 4.

GI Absorption	BBB Permeant	CYP1A2 Inhibitor	CYP2C19 Inhibitor	CYP2C9 Inhibitor	CYP2D6 Inhibitor	CYP3A4 Inhibitor	Lipinski
Low	No	No	No	No	No	No	Yes; 1 violation: NorO > 10

**Table 5:** In silico toxicity of compound 4.

hERG Blockers	H-HT	DILI	AMES Toxicity	Rat Oral Acute Toxicity	FDAMDD	Skin Sensitization	Carcinogenicity
0.01	0.742	0.908	0.034	0.035	0.004	0.082	0.152

## Data Availability

All data are available in the manuscript and Supplementary Materials.

## Supplementary Materials

The supporting information can be downloaded at: https://www.mdpi.com/article/10.3390/ijms24032270/s1.

## References

[1] Kirmani S.A.K., Ali P., Azam F. (2021). Topological Indices and QSPR/QSAR Analysis of Some Antiviral Drugs Being Investigated for the Treatment of COVID-19 Patients. Int. J. Quantum Chem..

[2] Tahir ul Qamar M., Zhu X.-T., Chen L.-L., Alhussain L., Alshiekheid M.A., Theyab A., Algahtani M. (2022). Target-Specific Machine Learning Scoring Function Improved Structure-Based Virtual Screening Performance for SARS-CoV-2 Drugs Development. Int. J. Mol. Sci..

[3] Mohammed H.A., Khan R.A., Abdel-Hafez A.A., Abdel-Aziz M., Ahmed E., Enany S., Mahgoub S., Al-Rugaie O., Alsharidah M., Aly M.S.A. (2021). Phytochemical Profiling, in Vitro and in Silico Anti-Microbial and Anti-Cancer Activity Evaluations and Staph GyraseB and h-TOP-IIβ Receptor-Docking Studies of Major Constituents of *Zygophyllum coccineum* L. Aqueous-Ethanolic Extract and Its Subsequent Fractions: An Approach to Validate Traditional Phytomedicinal Knowledge. Molecules.

[4] Mohammed H.A. (2020). The Valuable Impacts of Halophytic Genus Suaeda; Nutritional, Chemical, and Biological Values. Med. Chem..

[5] Khan R.A. (2018). Natural Products Chemistry: The Emerging Trends and Prospective Goals. Saudi Pharm. J..

[6] Alharbi S.A., AL-Juhani W.S., Albokhari E.J. (2022). Plastome Characterization, Phylogenetic Relationships, and Regional Conservation Status of *Ficus populifolia* Vahl. (Moraceae), a Peripherally Isolated Plant Population in the Arabian Peninsula. Forests.

[7] Alfarhan A.H., Al-Turki T.A., Basahy A.Y. (2005). Flora of Jizan Region.

[8] Bwalya A.G. (2015). Evaluation of the In Vitro Biological Activities and Phytochemical Profiling of Eight Ficus Species Collected in Zambia. Ph.D. Thesis.

[9] Odugbemi T. (2008). A Textbook of Medicinal Plants from Nigeria.

[10] Augustino S., Gillah P.R. (2005). Medicinal Plants in Urban Districts of Tanzania: Plants, Gender Roles and Sustainable Use. Int. For. Rev..

[11] Lansky E.P., Paavilainen H.M. (2010). Figs: The Genus Ficus.

[12] Deepa P., Sowndhararajan K., Kim S., Park S.J. (2018). A Role of Ficus Species in the Management of Diabetes Mellitus: A Review. J. Ethnopharmacol..

[13] Kaur A., Rana A.C., Tiwari V., Sharma R., Kumar S. (2011). Review on Ethanomedicinal and Pharmacological Properties of Ficus Religiosa. J. Appl. Pharm. Sci..

[14] Mousa O., Vuorela P., Kiviranta J., Wahab S.A., Hiltunen R., Vuorela H. (1994). Bioactivity of Certain Egyptian Ficus Species. J. Ethnopharmacol..

[15] Patil M.S., Patil C.R., Patil S.W., Jadhav R.B. (2011). Anticonvulsant Activity of Aqueous Root Extract of Ficus Religiosa. J. Ethnopharmacol..

[16] Gautam S., Meshram A., Bhagyawant S.S., Srivastava N. (2014). Ficus Religiosa-Potential Role in Pharmaceuticals. Int. J. Pharm. Sci. Res..

[17] Subramanian P.M., Misra G.S. (1978). Chemical Constituents of Ficus Bengalensis (Part II). Pol. J. Pharmacol. Pharm..

[18] Tsai P.-W., Castro-Cruz D., Shen C.-C., Chiou C.-T., Ragasa C.Y. (2012). Chemical Constituents of Ficus Odorata. Pharm. Chem. J..

[19] Almahy H.A., Rahmani M., Sukari M.A., Ali A.M. (2003). The Chemical Constituents of *Ficus benjamina* Linn. and Their Biological Activities. Pertanika J. Sci. Technol.

[20] Ragasa C.Y., Alimboyoguen A.B., Shen C.-C. (2014). Chemical Constituents of Ficus Nota. Der Pharma Chem..

[21] Vaya J., Mahmood S. (2006). Flavonoid Content in Leaf Extracts of the Fig (*Ficus carica* L.), Carob (*Ceratonia siliqua* L.) and Pistachio (*Pistacia lentiscus* L.). Biofactors.

[22] Kuete V., Ngameni B., Simo C.C.F., Tankeu R.K., Ngadjui B.T., Meyer J.J.M., Lall N., Kuiate J.R. (2008). Antimicrobial Activity of the Crude Extracts and Compounds from Ficus Chlamydocarpa and Ficus Cordata (Moraceae). J. Ethnopharmacol..

[23] Raga D.D., Cheng C.L.C., Lee K.C.I.C., Olaziman W.Z.P., De Guzman V.J.A., Shen C.-C. (2011). Bioactivities of Triterpenes and a Sterol from Syzygium Samarangense. Z. Für Naturforsch. C.

[24] Dembele O., Haidara M., Ndong A., Barbosa F., Sy G.Y., Wélé A. (2022). Potential Analgesic Activity of the Methanolic F4 Fraction of Leaf Extracts of Annona Senegalensis Pers. (Annonaceae). J. Drug Deliv. Ther..

[25] Mohammed H.A., Al-Omar M.S., Mohammed S.A.A., Alhowail A.H., Eldeeb H.M., Sajid M.S.M., Abd-Elmoniem E.M., Alghulayqeh O.A., Kandil Y.I., Khan R.A. (2021). Phytochemical Analysis, Pharmacological and Safety Evaluations of Halophytic Plant, *Salsola cyclophylla*. Molecules.

[26] Kariuki D.K., Kanui T.I., Mbugua P.M., Githinji C.G. (2012). Analgesic and Anti-Inflammatory Activities of 9-Hexacosene and Stigmasterol Isolated from *Mondia whytei*. Phytopharmacology.

[27] Nirmal S.A., Pal S.C., Mandal S.C., Patil A.N. (2012). Analgesic and Anti-Inflammatory Activity of β-Sitosterol Isolated from Nyctanthes Arbortristis Leaves. Inflammopharmacology.

[28] Ahmed F., Rahman M.Z., Khan M.F., Rashid M.A., Rahman M.S. (2019). β-Amyrin as an Analgesic Component of the Leaves of *Callistemon citrinus* (Curtis) Skeels: Chemical, Biological and in Silico Studies. Bangladesh J. Bot..

[29] Jannat T., Hossain M.J., El-Shehawi A.M., Kuddus M.R., Rashid M.A., Albogami S., Jafri I., El-Shazly M., Haque M.R. (2022). Chemical and Pharmacological Profiling of *Wrightia coccinea* (Roxb. Ex Hornem.) Sims Focusing Antioxidant, Cytotoxic, Antidiarrheal, Hypoglycemic, and Analgesic Properties. Molecules.

[30] Thamaraiselvi L., Selvankumar T., Wesely E.G., Nathan V.K. (2021). In-Silico Molecular Docking Analysis of Some Plant Derived Molecules for Anti-Inflammatory Inhibitory Activity. Curr. Bot.

[31] Akinloye O.A., Akinloye D.I., Onigbinde S.B., Metibemu D.S. (2020). Phytosterols Demonstrate Selective Inhibition of COX-2: In-Vivo and In-Silico Studies of Nicotiana Tabacum. Bioorg. Chem..

[32] Noble F., Smadja C., Valverde O., Maldonado R., Coric P., Turcaud S., Fournié-Zaluski M.-C., Roques B.P. (1997). Pain-Suppressive Effects on Various Nociceptive Stimuli (Thermal, Chemical, Electrical and Inflammatory) of the First Orally Active Enkephalin-Metabolizing Enzyme Inhibitor RB 120. Pain.

[33] Lanhers M.-C., Fleurentin J., Mortier F., Vinche A., Younos C. (1992). Anti-Inflammatory and Analgesic Effects of an Aqueous Extract of *Harpagophytum procumbens*. Planta Med..

[34] Bertram G.K. (1998). Basic and Clinical Pharmacology: Appleton and Lange. Stamford Connect..

[35] Lee Y., Rodriguez C., Dionne R.A. (2005). The Role of COX-2 in Acute Pain and the Use of Selective COX-2 Inhibitors for Acute Pain Relief. Curr. Pharm. Des..

[36] Sadik O.A., Yazgan I., Eroglu O., Liu P., Olsen S.T., Moser A.M., Sander P.G., Tsiagbe C., Harada K., Bajwa S. (2018). Objective Clinical Pain Analysis Using Serum Cyclooxygenase-2 and Inducible Nitric Oxide Synthase in American Patients. Clin. Chim. Acta.

[37] Lenherr A., Mabry T.J. (1987). Acetylated Allose-Containing Flavonoid Glucosides from *Stachys anisochila*. Phytochemistry.

[38] Lenherr A., Lahloub M.F., Sticher O. (1984). Three Flavonoid Glycosides Containing Acetylated Allose from *Stachys recta*. Phytochemistry.

[39] Hui Y., Shuang-Xi M.E.I., Li-Yan P., Zhong-Wen L.I.N., Han-Dong S.U.N. (2002). A New Glucoside from *Rhodiola fastigiata* (Crassulaceae). J. Integr. Plant Biol..

[40] Della Greca M., Monaco P., Previtera L. (1990). Stigmasterols from Typha Latifolia. J. Nat. Prod..

[41] Mohammed H.A., Abdelwahab M.F., El-Ghaly E.-S.M., Ragab E.A. (2021). Phytochemical Characterization, In Vitro Anti-Inflammatory, Anti-Diabetic, and Cytotoxic Activities of the Edible Aromatic Plant; *Pulicaria jaubertii*. Molecules.

[42] Gupta R., Sharma A.K., Dobhal M.P., Sharma M.C., Gupta R.S. (2011). Antidiabetic and Antioxidant Potential of Β-Sitosterol in Streptozotocin-Induced Experimental Hyperglycemia. J. Diabetes.

[43] Mohammed H.A., Al-Omar M.S., El-Readi M.Z., Alhowail A.H., Aldubayan M.A., Abdellatif A.A.H. (2019). Formulation of Ethyl Cellulose Microparticles Incorporated Pheophytin A Isolated from Suaeda Vermiculata for Antioxidant and Cytotoxic Activities. Molecules.

[44] Knight S.A. (1974). Carbon-13 NMR Spectra of Some Tetra- and Pentacyclic Triterpenoids. Org. Magn. Reson..

[45] Orlando B.J., Malkowski M.G. (2016). Substrate-Selective Inhibition of Cyclooxygeanse-2 by Fenamic Acid Derivatives Is Dependent on Peroxide Tone. J. Biol. Chem..

[46] Daina A., Michielin O., Zoete V. (2017). SwissADME: A Free Web Tool to Evaluate Pharmacokinetics, Drug-Likeness and Medicinal Chemistry Friendliness of Small Molecules. Sci. Rep..

[47] Balanean L., Braicu C., Berindan-Neagoe I., Nastasa C., Tiperciuc B., Verite P., Oniga O. (2014). Synthesis of Novel 2-Metylamino-4-Substituted-1, 3-Thiazoles with Antiproliferative Activity. Rev. Chim..

[48] Ertl P., Rohde B., Selzer P. (2000). Fast Calculation of Molecular Polar Surface Area as a Sum of Fragment-Based Contributions and Its Application to the Prediction of Drug Transport Properties. J. Med. Chem..

[49] Hughes J.D., Blagg J., Price D.A., Bailey S., DeCrescenzo G.A., Devraj R.V., Ellsworth E., Fobian Y.M., Gibbs M.E., Gilles R.W. (2008). Physiochemical Drug Properties Associated with in Vivo Toxicological Outcomes. Bioorg. Med. Chem. Lett..

[50] Jain A.K., Singh D., Dubey K., Maurya R., Mittal S., Pandey A.K. (2018). Models and Methods for In Vitro Toxicity. In Vitro Toxicology.

[51] Lee H.M., Yu M.S., Kazmi S.R., Oh S.Y., Rhee K.H., Bae M.A., Lee B.H., Shin D.S., Oh K.S., Ceong H. (2019). Computational Determination of HERG-Related Cardiotoxicity of Drug Candidates. BMC Bioinform..

[52] Nohmi T. (2018). Thresholds of Genotoxic and Non-Genotoxic Carcinogens. Toxicol. Res..

[53] Prakash V.E.D. (2017). Terpenoids as Source of Anti-Inflammatory Compounds. Asian J. Pharm. Clin. Res..

[54] Saudagar R.B., Saokar S. (2019). Anti-Inflammatory Natural Compounds from Herbal and Marine Origin. J. Drug Deliv. Ther..

[55] Williamson E.M., Okpako D.T., Evans F.J. (1996). Selection, Preparation and Pharmacological Evaluation of Plant Material, Volume 1.

[56] Koster R. (1959). Acetic Acid for Analgesic Screening. Fed. Proc..

[57] Gulecha V., Sivakumar T., Upaganlawar A., Mahajan M., Upasani C. (2011). Screening of Ficus Religiosa Leaves Fractions for Analgesic and Anti-Inflammatory Activities. Indian J. Pharmacol..

[58] Liao C.-R., Kao C.-P., Peng W.-H., Chang Y.-S., Lai S.-C., Ho Y.-L. (2012). Analgesic and Anti-Inflammatory Activities of Methanol Extract of *Ficus pumila* L. in Mice. Evid.-Based Complement. Altern. Med..

[59] Das P.C., Das A., Mandal S. (1989). Antimicrobial and Anti-Inflammatory Activities of the Seed Kernel of *Mangifera indica*. Fitoterapia.

[60] Otimenyin S.O., Uguru M.O., Atang B.L. (2004). Antiinflamatory and Analgesic Activities of Ficus Thonningii and Pseudocedrela Kotschyi Extracts. Niger. J. Pharm. Res..

[61] Abdulmalik I.A., Sule M.I., Yaro A.H., Abdullahi M.I., Abdulkadir M.E., Yusuf H. (2011). Evaluation of Analgesic and Anti-Inflammatory Effects of Ethanol Extract of *Ficus iteophylla* Leaves in Rodents. Afr. J. Tradit. Complement. Altern. Med..

[62] Chemical Computing Group (2016). Molecular Operating Environment (MOE).

