# Supplementary material for: In Vivo and In Silico Analgesic Activity of Ficus populifolia Extract Containing 2-O-β-D-(3′,4′,6′-Tri-acetyl)-glucopyranosyl-3-methyl Pentanoic Acid
Source: Int J Mol Sci. 2023 Jan 23;24(3):2270. doi: 10.3390/ijms24032270 (PMC9916429; doi:10.3390/ijms24032270)
Supplement: Supplementary file 1 [file ijms-24-02270-s001.zip › ijms-2135393-supplementary.pdf]

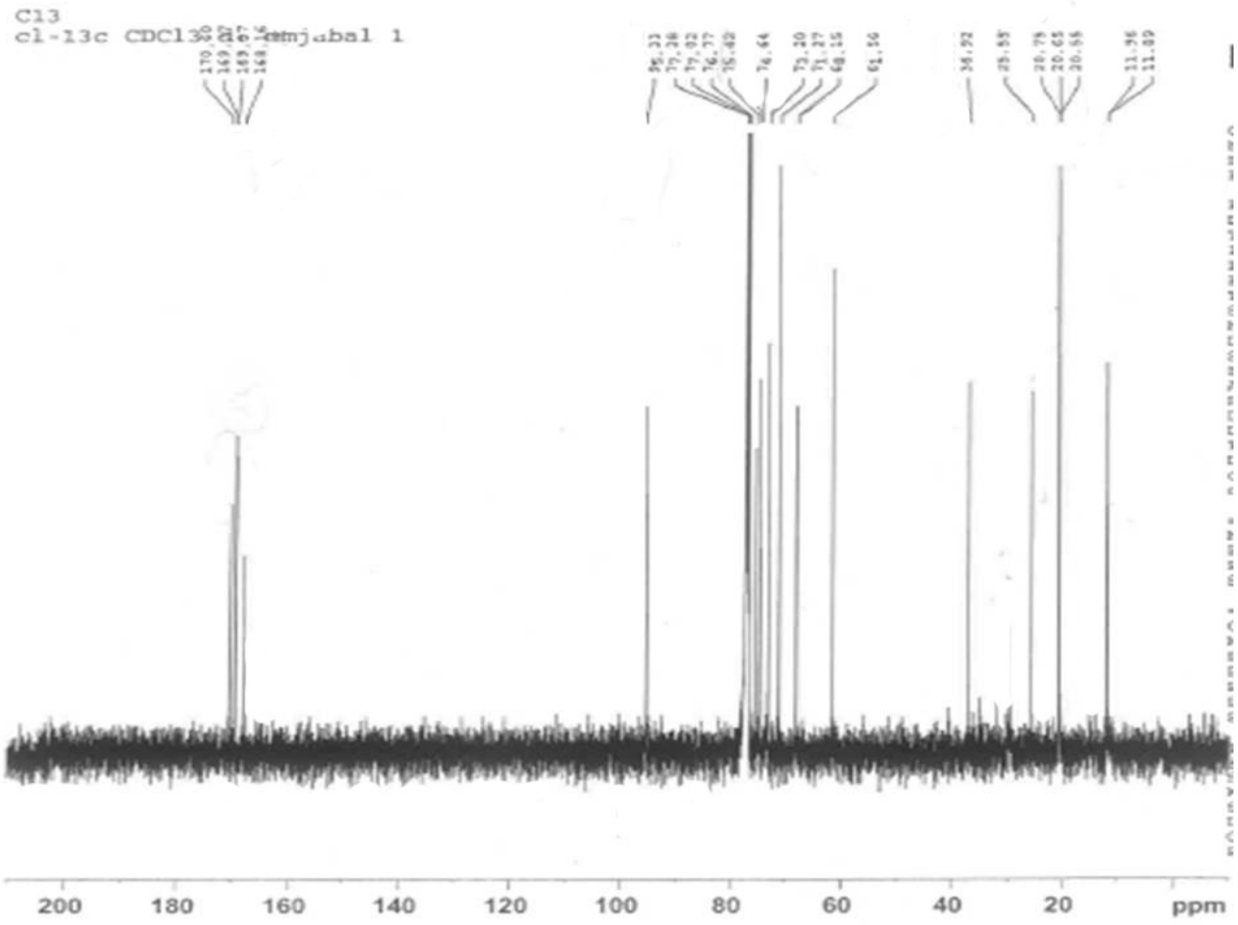

Figure S1. ^{13}C-NMR spectrum of compound 4.

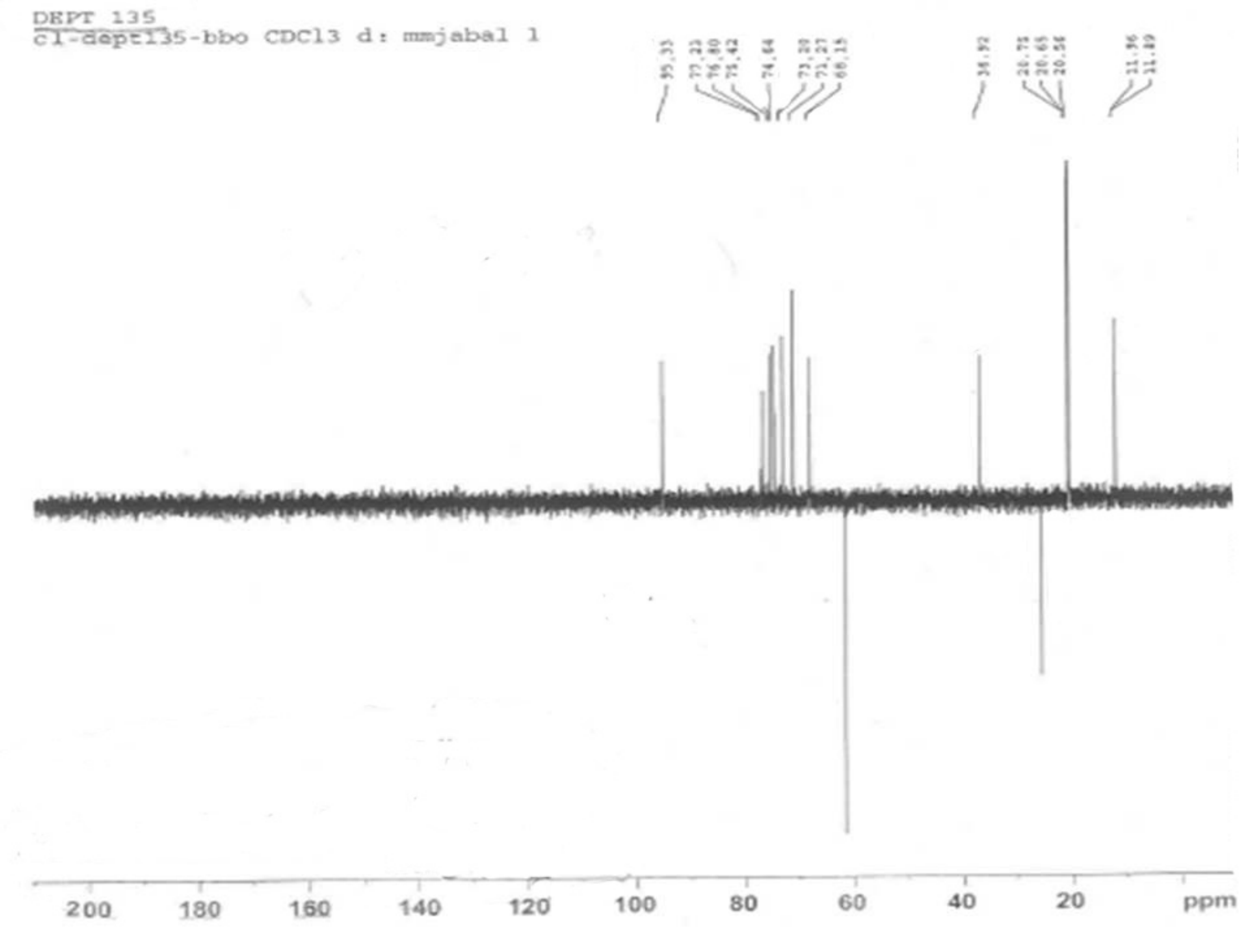

Figure S2. DEPT-135 spectrum of compound 4.

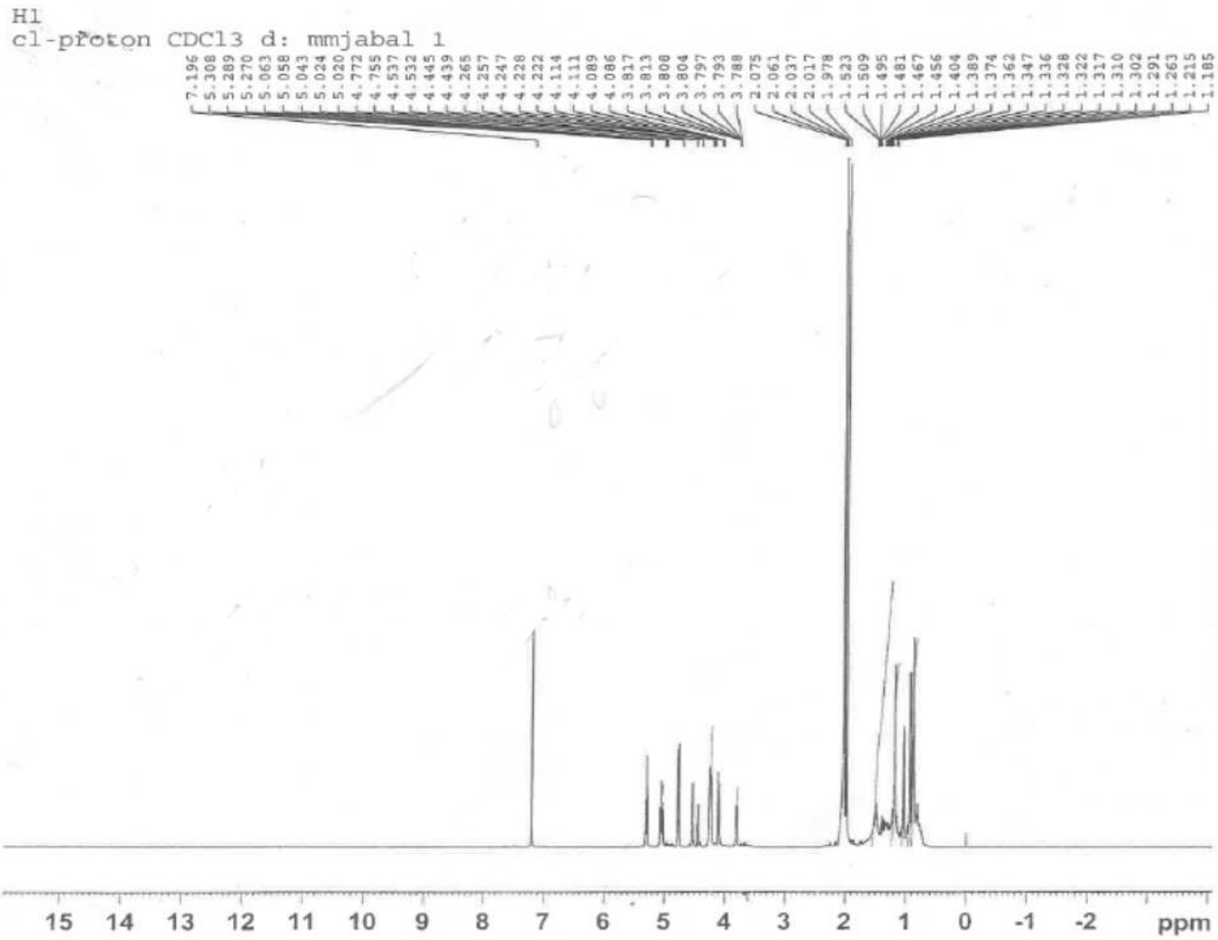

Figure S3. ^{1}H-NMR spectrum of Compound 4.

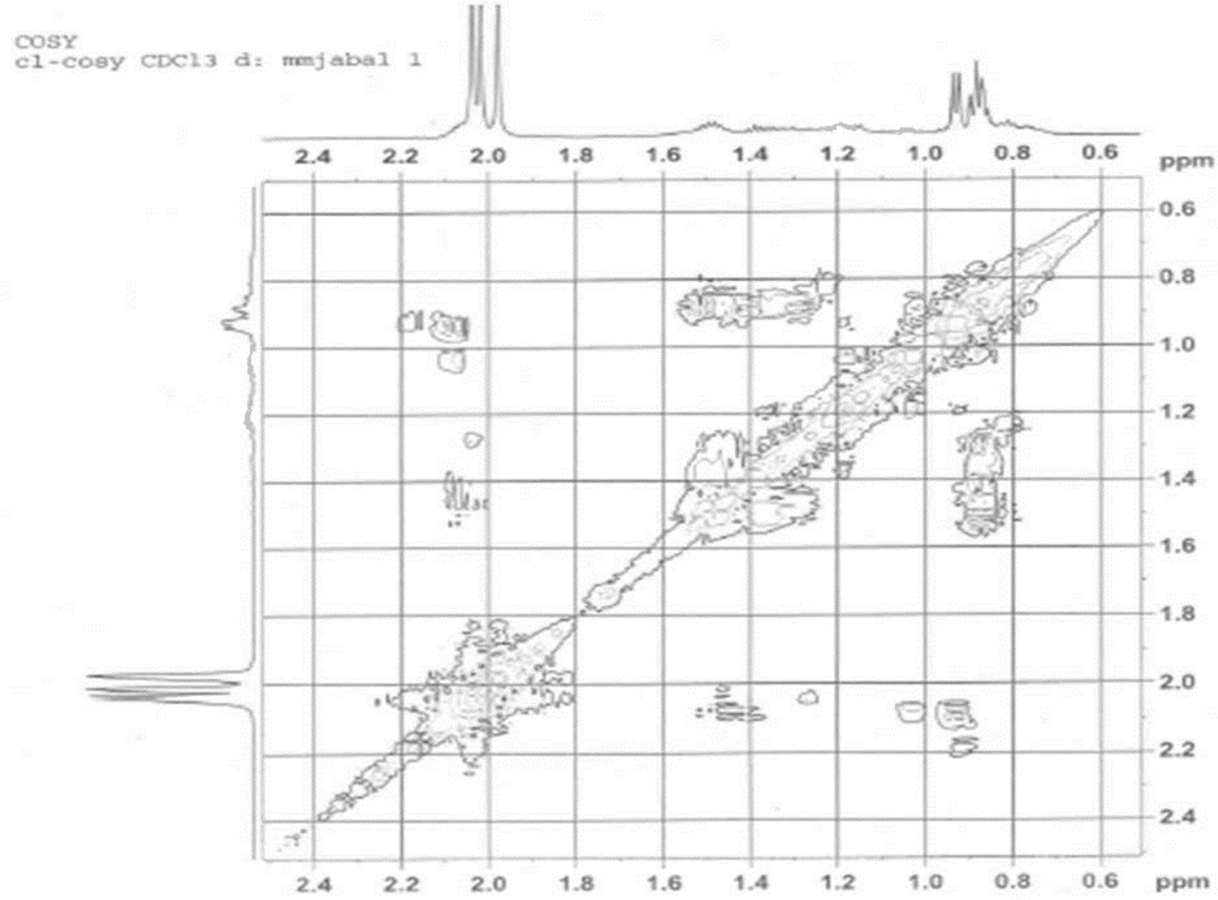

Figure S4. H-H COSY spectrum of compound 4.

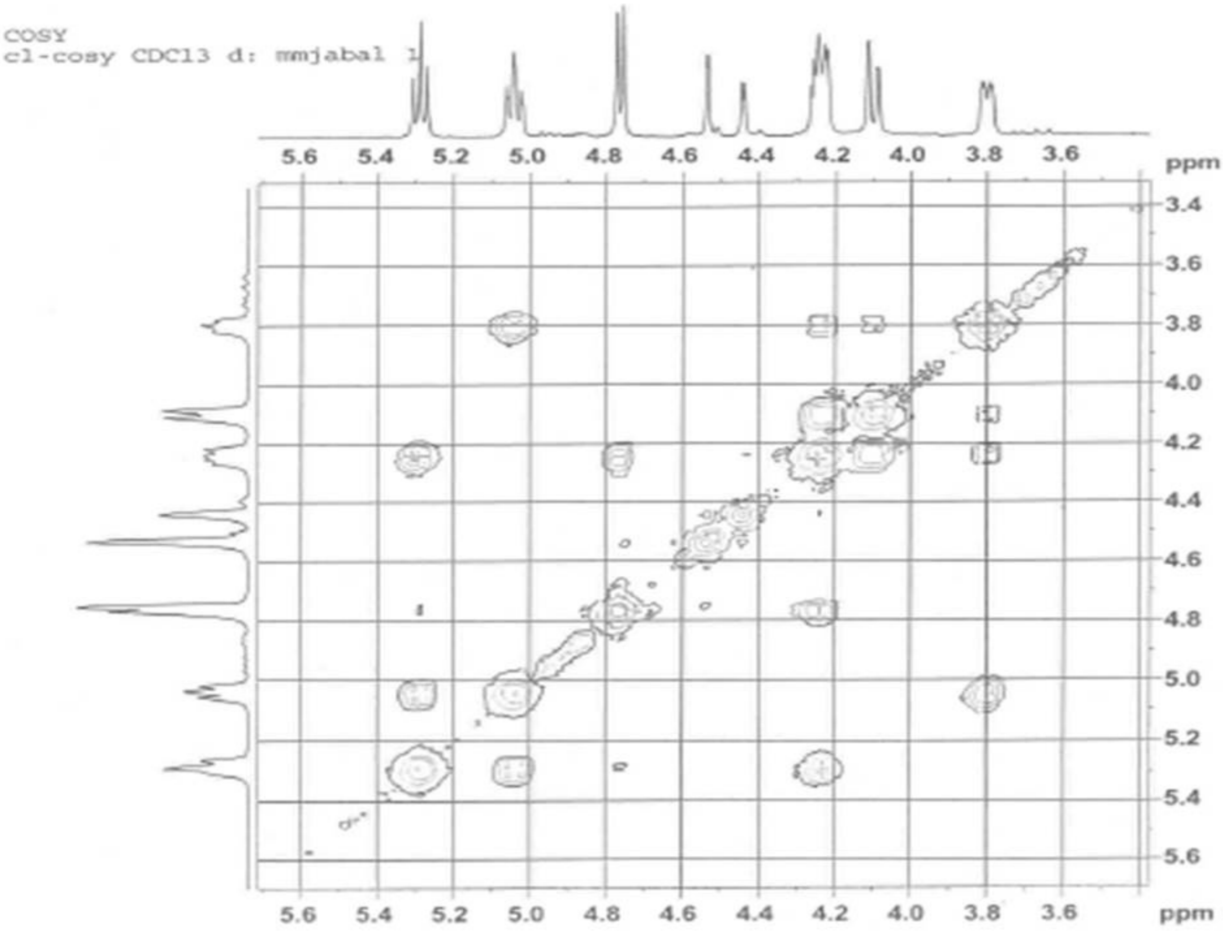

Figure S5. Expanded COSY spectrum of compound 4.

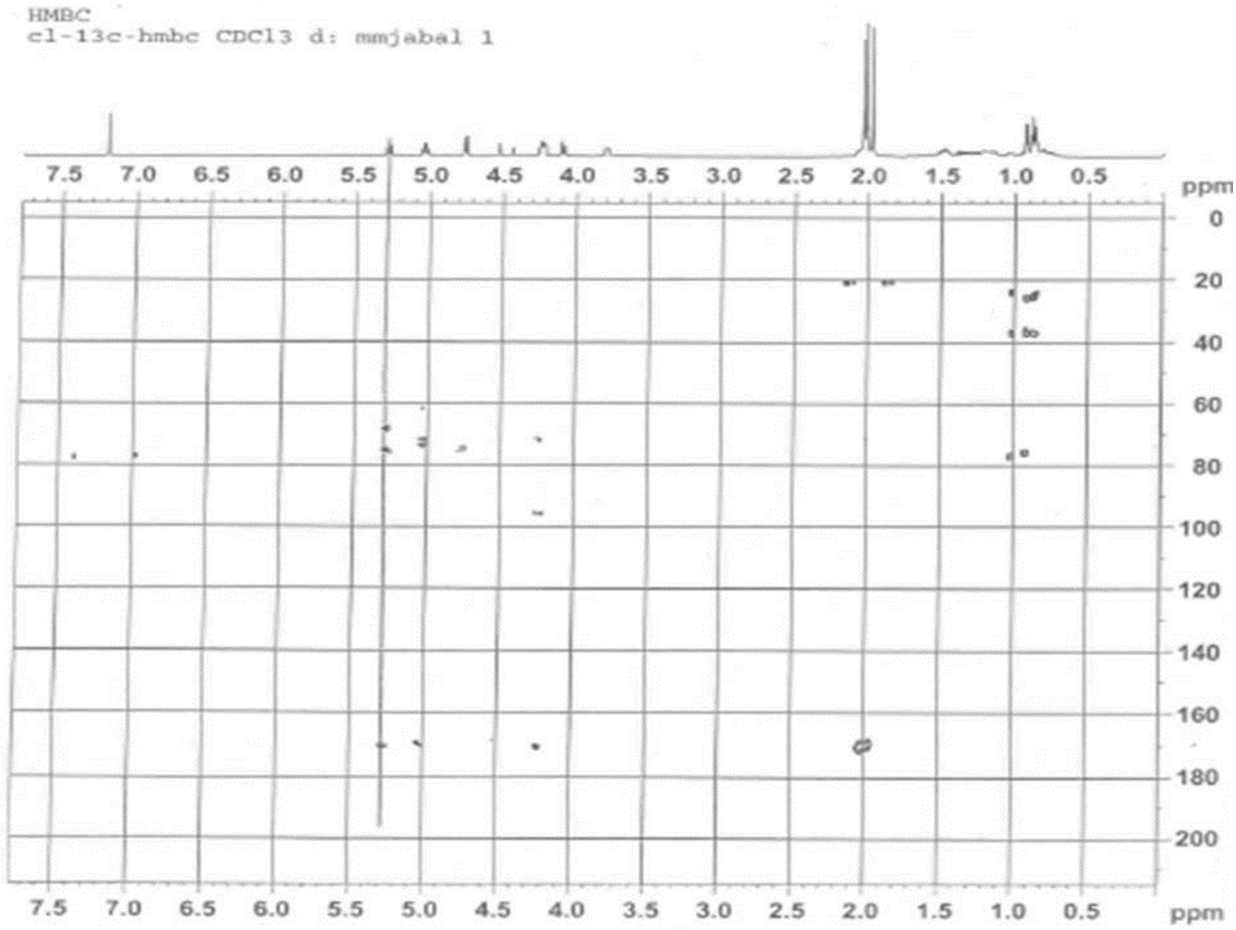

Figure S6. HMBC spectrum of compound 4.
